# Morphological and life‐history trait plasticity of two *Daphnia* species induced by fish kairomones

**DOI:** 10.1002/ece3.11422

**Published:** 2024-06-06

**Authors:** Qide Jin, Yeping Wang, Kun Zhang, Guoqing Li, Yanan Chen, Yujuan Hong, Hanxue Cheng, Daogui Deng

**Affiliations:** ^1^ School of Life Sciences Huaibei Normal University Huaibei Anhui China

**Keywords:** *Aristichthys nobilis*, *Daphnia*, inducible defenses, kairomone, phenotypic plasticity

## Abstract

*Daphnia* can avoid predation by sensing fish kairomones and producing inducible defenses by altering the phenotype. In this study, the results showed that the morphological and life‐history strategies of two *Daphnia* species (*Daphnia pulex* and *Daphnia sinensis*) exposed to *Aristichthys nobilis* kairomones. In the presence of fish kairomones, the two *Daphnia* species exhibited significantly smaller body length at maturity, smaller body length of offspring at the 10th instar, and longer relative tail spine of offspring. Nevertheless, other morphological and life‐history traits of the two *Daphnia* species differed. *D. pulex* showed a significantly longer relative tail spine length and earlier age at maturity after exposure to fish kairomones. The total offspring number of *D. sinensis* exposed to fish kairomones was significantly higher than that of the control group, whereas that of *D. pulex* was significantly lower. These results suggest that the two *Daphnia* species have different inducible defense strategies (e.g., morphological and life‐history traits) during prolonged exposure to *A. nobilis* kairomones, and their offspring also develop morphological defenses to avoid predation. It will provide reference for further exploring the adaptive evolution of *Daphnia* morphology and life‐history traits in the presence of planktivorous fish.

## Graphical abstract

This study investigated the morphological and life‐history trait plasticity of two *Daphnia* species (*Daphnia pulex* and *Daphnia sinensis*) induced by fish kairomones. The inducible mechanisms of the two *Daphnia* species exposed to fish kairomones differ significantly, and their offspring also develop morphological defenses to reduce the risk of fish predation.

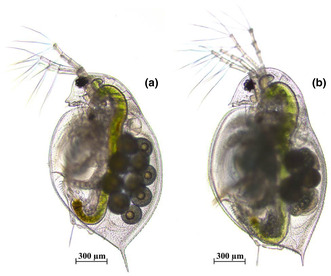

## 1 INTRODUCTION

Phenotypic plasticity is the ability of organisms to express different phenotypes depending on their biotic and abiotic environments (Agrawal, 2001). It is an effective way to improve the fitness of organisms in variable environments (Dodson, 1989; Stearns, 1989). Predation is a major factor controlling the adaptation of organisms to environmental changes (Weiss, 2019) and can promote natural selection within populations (Kuchta & Svensson, 2014; Morgans & Ord, 2013) and maintain species diversity (Fine, 2015). In general, organisms can develop effective strategies (e.g., constitutive or inducible defenses) to avoid predation (De Alkimin et al., 2020; Harvell, 1990; Ritschar et al., 2020).

Predator‐induced defenses represent intriguing forms of phenotypic plasticity that can not only decrease the possibility of encountering predators and effectively reduce predator attacks, but also reduce the chances of consuming the prey after predators attack (Dodson, 1974; Kolar & Wahl, 1998; Weiss, 2019). Generally, chemical cues (e.g., kairomones) related to inducible defenses are unintentionally released by predators, signaling their existence to the prey (Diel et al., 2020). *Daphnia* are found in freshwater ecosystems and are regarded as model organisms for studying the phenotypic plasticity of zooplankton in response to kairomones release by predators (Dodson, 1989; Seda & Petrusek, 2011; Weetman & Atkinson, 2002). Kairomones released by predators can induce changes in the morphology, behavior, and life‐history traits of *Daphnia*, thereby affecting their community structures and food webs (Hoverman et al., 2005; Werner & Peacor, 2003).

*Daphnia* exhibit different patterns of morphological plasticity when exposed to kairomones. Fish kairomones often reduce the body size at first reproduction of *Daphnia* (Beklioglu et al., 2010; Hanazato et al., 2001; Mikulski, 2001), and induce *Daphnia* to produce smaller offspring (Mikulski, 2001). In contrast, Hanazato et al. (2001) observed that *D. pulex* exposed to fish kairomones (*Pseudorasbora parva* and *Lepomis macrochirus*) produced larger offspring. Invertebrate predator (*Chaoborus* larvae) kairomones induced *D. pulex* to have a shorter body length at maturity and produce larger offspring (Riessen, 2012); however, this species exhibits a longer body length at first reproduction (Repka & Walls, 1998; Walls et al., 1997). Declerck and Weber (2003) also observed that *Daphnia galeata* has a longer body length at first reproduction when exposed to *Chaoborus* larva kairomones. Additionally, Sperfeld et al. (2020) reported that *Daphnia longispina* develops necktooth structures and has longer tail spines due to exposure to *Chaoborus* larva kairomones. Similarly, kairomones released by *Triops cancriformis* also induce longer tail spines in *Daphnia similis* (Ritschar et al., 2020). Furthermore, *Daphnia lumholtzi* offspring at first reproduction exposed to *Lepomis* kairomones has significantly longer head and tail spines compared with those of control group (Dzialowski et al., 2003). In several large Chinese lakes where vertebrate and invertebrate predators coexist, *D. sinensis* has prominent recurvate helmets, longer relative helmet lengths, and smaller sizes (Ma et al., 2016). These results indicate that the effects of predator kairomones on the morphological plasticity of *Daphnia* are complex and vary with different predators and prey.

*Aristichthys nobilis* is a typical planktivorous fish that mainly feeds on zooplankton (Ni & Jiang, 1954) and is distributed in rivers, lakes, and reservoirs in China (Chen, 1998). *A. nobilis* plays an important role in regulating the water quality (Chen et al., 2007) and maintaining the balance of aquatic ecosystems (Yang et al., 2013). The filter‐feeding *Hypophthalmichthys molitrix* and *A. nobilis* could graze on cyanobacteria and control cyanobacterial blooms in lakes (Liu & Xie, 1999; Xie & Liu, 2001), and *A. nobilis* mainly feed on zooplankton. Lake Chaohu, one of the five largest freshwater lakes in China, is used for irrigation, water supply, and fishing (Wang & Dou, 1998). To control the frequent occurrence of cyanobacterial blooms in Lake Chaohu, *A. nobilis* and *H. molitrix* have been continuously released into the lake since 1991 (Li, 2017; Lv et al., 2011). Both *D. sinensis* and *D. pulex* have been identified in Lake Chaohu, and *D. pulex* dominate at lower temperatures (March and April) whereas *D. sinensis* are dominant at higher temperatures (May and June) (Deng et al., 2008; Xu et al., 2014). However, the effect of *A. nobilis* on the morphological plasticity and life‐history traits of these two *Daphnia* species remains unclear.

In this study, we investigated the morphological and life‐history changes in the two *Daphnia* species (*D. pulex* and *D. sinensis*, with different niche characteristics in Lake Chaohu) exposed to two *A. nobilis* kairomone concentrations (and control). Our goals were to explore the inducible strategies of the two *Daphnia* species in response to *A. nobilis* kairomones at different growth stages, and to discuss the reasons for the inducible defense changes of the two *Daphnia* species during prolonged exposure to *A. nobilis* kairomone. We hypothesized that in the presence of *A. nobilis* kairomones, *D. pulex* and *D. sinensis* might form different induced defense strategies (morphological and life‐history defenses) by regulating the growth or reproduction resources, and their offspring might also develop morphological defenses to reduce the predation risk.

## 2 MATERIALS AND METHODS

### 2.1 *Tetradesmus obliquus* and *Daphnia* culture

*Tetradesmus obliquus* was purchased from the Freshwater Algae Culture Collection at the Institute of Hydrobiology, The Chinese Academy of Sciences (Wuhan City, China). *T. obliquus* was cultured in BG‐11 medium at 25 ± 1°C, with a light intensity of 2200 lx and a light–dark photoperiod of 12:12 h. *T. obliquus* were collected and stored in a refrigerator at 4°C when they reached the exponential growth period.

**FIGURE 1:**
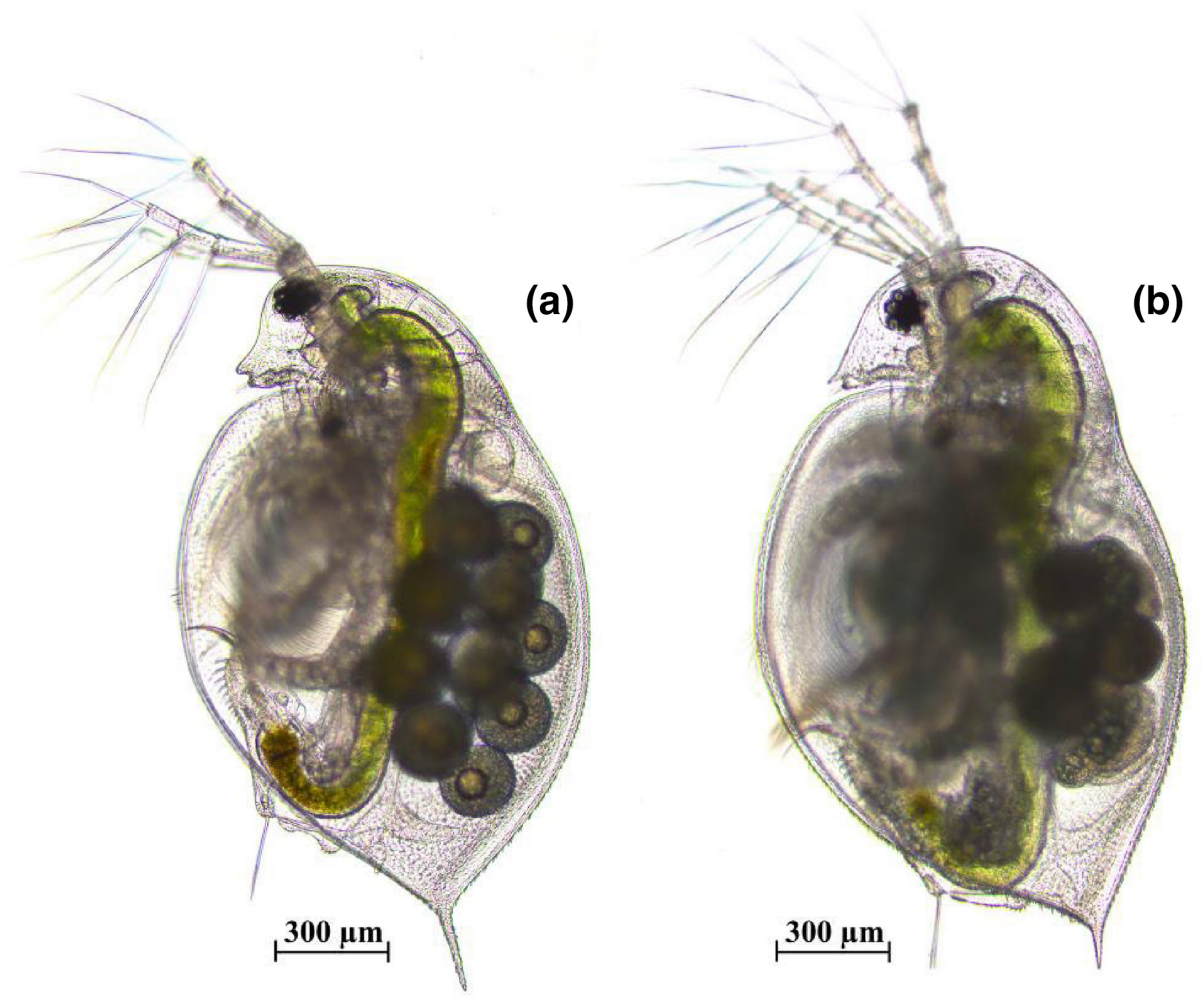
The photograph of *Daphnia pulex* female (a) and *Daphnia sinensis* female (b).

Two *Daphnia* species (*D. pulex* and *D. sinensis*, Figure 1) were hatched from resting eggs collected from Lake Chaohu (Anhui Province, China). One female adult was selected from each *Daphnia* species as the grandmother and incubated in a 50‐mL beaker containing 40 mL culture solution at (25 ± 1)°C, with a light intensity of 2200 lx and a light–dark photoperiod of 12:12 h. The culture solution is tap water that has been aerated for more than 48 h and filtered according to the method of Peng et al. (2018). The offspring produced by the grandmother (the mothers) was cultured under the same conditions in a 50‐mL beaker at (25 ± 1)°C. Third‐generation offspring (birth time <6 h) produced by the mothers was regarded as experimental animals. Green algae (*T. obliquus*) at a biomass of 3.5 mg C/L were used as a food source of experimental animals.

### 2.2 Fish kairomone acquisition

A *A. nobilis* individual (body length: 49 cm, body width: 11 cm, body weight: 1.32 kg, age: about 1 year old) was placed in 30 L of tap water that had aerated for more than 48 h. After 24 h of starvation, the tap water was replaced, and *A. nobilis* was cultured for 24 h under the same conditions. In order to remove feces from *A. nobilis* and large particulate matters, water‐bearing fish kairomones were first filtered with a 100‐mesh sieve and filter papers (9 cm, 20 μm pore size). Then, they were filtered again using GF/F Whatman™ filters (47 mm, 0.7 μm pore size) to remove smaller particulate matters. Finally, filtered fish‐incubation water was used as the source of *A. nobilis* kairomones in the experiments. Fish kairomone acquisition has referred to previous studies (Cao et al., 2023; Hahn et al., 2019). The fish‐incubation water was transferred to sealed bags and stored at −20°C (Cao et al., 2023; Lv et al., 2023; Pestana et al., 2013).

### 2.3 Experiment design

Three groups (one control group and two fish kairomone concentration groups) were established: C group (control), 100% culture solution; F_10_ group, 10% fish‐incubation water +90% culture solution; and F_20_ group, 20% fish‐incubation water +80% culture solution. The culture solution is tap water that has been aerated for more than 48 h and filtered. Each group consisted of 15 experimental animals for each *Daphnia* species. Each experimental animal (birth time < 6 h) was placed in a 50‐mL beaker containing 40 mL of culture solution (for the control group) or fish‐incubation water + culture solution (for fish kairomone concentration groups). The experiment was conducted at (25 ± 1)°C, with a light intensity of 2200 lx and a light–dark photoperiod of 12:12 h. The experimental animals were transferred into fresh medium (culture solution or fish‐incubation water + culture solution) every day, and *T. obliquus* (3.5 mg C/L) as food of the experimental animals was replaced at the same time. The newborns produced by two *Daphnia* species were promptly removed from the beakers during the experiment period. The experiments were terminated at the end of the 10th instar of the two *Daphnia* species. The body and tail spine lengths of the two *Daphnia* species were measured using a microscope (Olympus CX21), and the number of eggs and offspring and their age at maturity were recorded. Additionally, 50 offspring produced by each *Daphnia* species at first reproduction and 10th instar were selected, and their body and tail spine lengths were measured.

### 2.4 Data analysis

The relative tail spine length was calculated as the ratio of the tail spine length to the body length of the two *Daphnia* species and their offspring. The growth rate of individual (μg/day) was calculated by the following formula: GR = (ln *W*_*t*_ − ln *W*_0_)/*t*; where *W* is the individual dry weight (μg), *t* is the number of days at the 10th instar, and *W*_*t*_ is the individual dry weight at the 10th instar, *W*_0_ is the individual dry weight at the beginning of the experiment (Qin et al., 2022). The dry weight of the two *Daphnia* was estimated based on the method of length–weight relationships (Zhang & Huang, 1991). The intrinsic rate of the increase (*r*) was calculated according to the Euler equation (Weber, 2003): 1 = Σe^−*rx*^ *l*_*x*_ *m*_*x*_, where *r* is the intrinsic rate of the increase for the population (/day), *x* is the age class (0, 1, … *N*), *l*_*x*_ is the probability of surviving to age *x*, and *m*_*x*_ is the fecundity at age *x*.

SPSS software (version 20.0) was used for the data analysis. Significant effects of fish kairomones on the morphology and life‐history traits of the two *Daphnia* species were detected using one‐way ANOVA, and differences among different *A. nobilis* kairomone concentration groups were analyzed using Tukey's HSD test. All data were transformed using ln (1 + *x*) before statistics to data standardization.

## 3 RESULTS

### 3.1 Effects of fish kairomones on the morphology of the two *Daphnia* species

**FIGURE 2:**
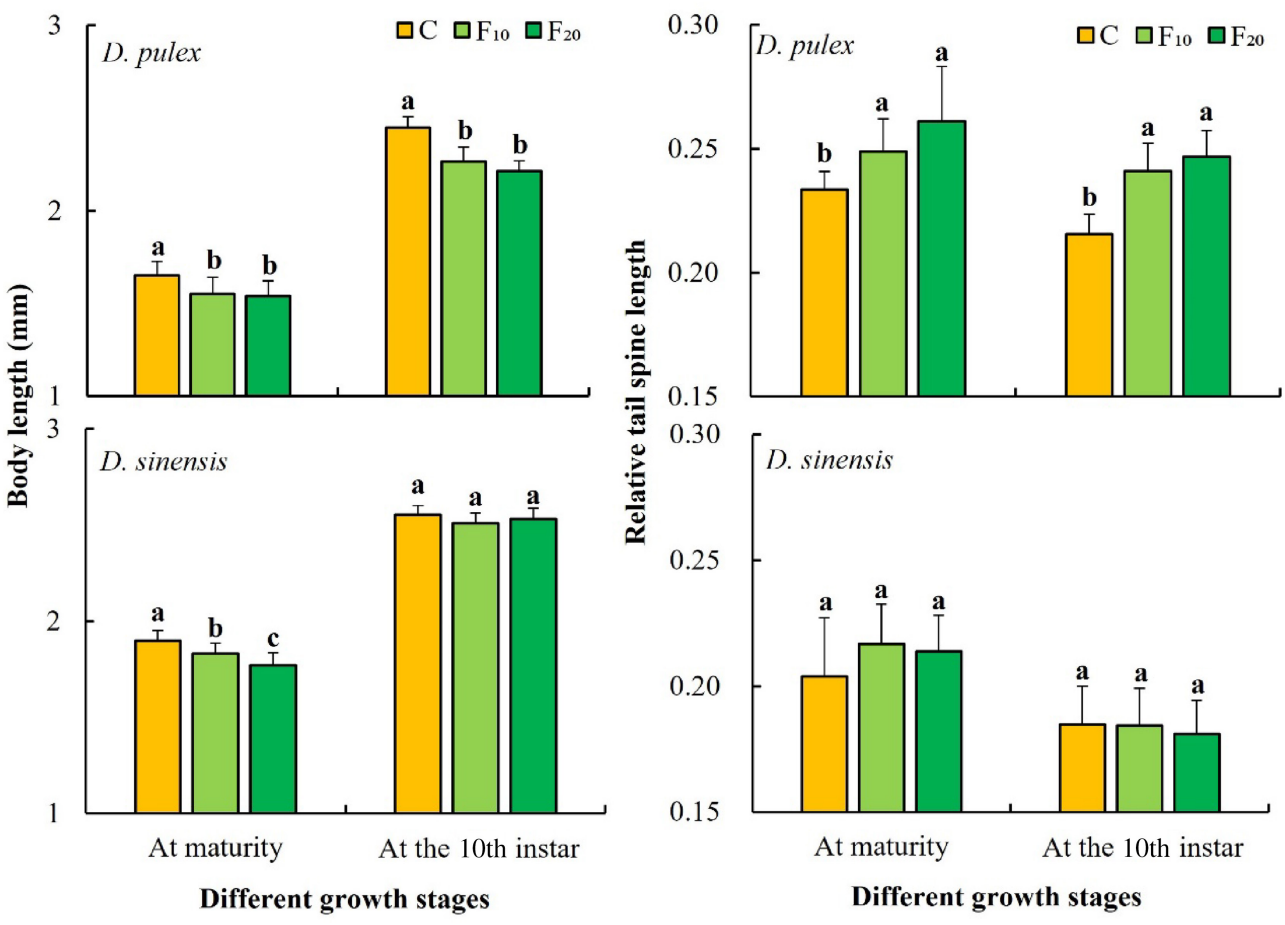
Body lengths and relative tail spine lengths of the two *Daphnia* species at different fish kairomone concentrations. Different letters indicate the significant differences (*p* < .05).

One‐way ANOVA showed that fish kairomones had significant effects on the body length at maturity of *D. pulex* and *D. sinensis* and also significantly affected the body length at the 10th instar of *D. pulex* (Table 1). The body lengths at maturity (*p* < .05) and in the 10th instar stage (*p* < .001) of *D. pulex* in the C group (control) were significantly longer than those in the F_10_ and F_20_ groups. The body length at maturity of *D. sinensis* in the C group (control) was significantly longer than that in the F_10_ (*p* < .05) and F_20_ (*p* < .001) groups, and it was significantly longer body length at maturity in the F_10_ group than the F_20_ group (*p* < .05). No significant differences were observed for the body length at the 10th instar of *D. sinensis* among the three groups (Figure 2).

**TABLE 1:** One‐way ANOVA results showing the effects of fish kairomones on the morphology and life‐history traits of the two *Daphnia* species.

Parameter	df	*Daphnia pulex*		*Daphnia sinensis*	
		*F*	*p*	*F*	*p*
Body length at maturity	2	7.499	**.002**	18.165	**<.001**
Relative tail spine length at maturity	2	11.066	**<.001**	1.865	.167
Growth rate at maturity	2	0.216	.807	8.871	**.001**
Age at maturity	2	3.377	**.044**	2.583	.087
Number of eggs at first pregnancy	2	2.774	.074	1.257	.295
Number of offspring at first reproduction	2	0.126	.882	1.430	.251
Body length of offspring at first reproduction	2	0.240	.787	12.172	**<.001**
Relative tail spine length of offspring at first reproduction	2	16.765	**<.001**	13.642	**<.001**
Body length at the 10th instar	2	47.378	**<.001**	2.448	.099
Relative tail spine length at the 10th instar	2	21.637	**<.001**	0.156	.856
Growth rate at the 10th instar	2	0.808	.453	1.047	.360
Body length of offspring at the 10th instar	2	31.960	**<.001**	22.460	**<.001**
Relative tail spine length of offspring at the 10th instar	2	6.498	**.002**	56.897	**<.001**
Total eggs number	2	4.612	**.015**	55.324	**<.001**
Total offspring number	2	11.693	**<.001**	33.456	**<.001**

*Note:* Bold values stand for significant effects.

One‐way ANOVA showed that fish kairomones had significant effects on the relative tail spine lengths at maturity and at the 10th instar of *D. pulex* but had no significant effects on those of *D. sinensis* (Table 1). The relative tail spine lengths at maturity and at the 10th instar of *D. pulex* in the C group (control) were significantly lower than those in the F_10_ (at maturity: *p* < .05; at the 10th instar: *p* < .001) and F_20_ groups (*p* < .001), whereas those of *D. sinensis* did not significantly differ among the three groups (Figure 2).

**FIGURE 3:**
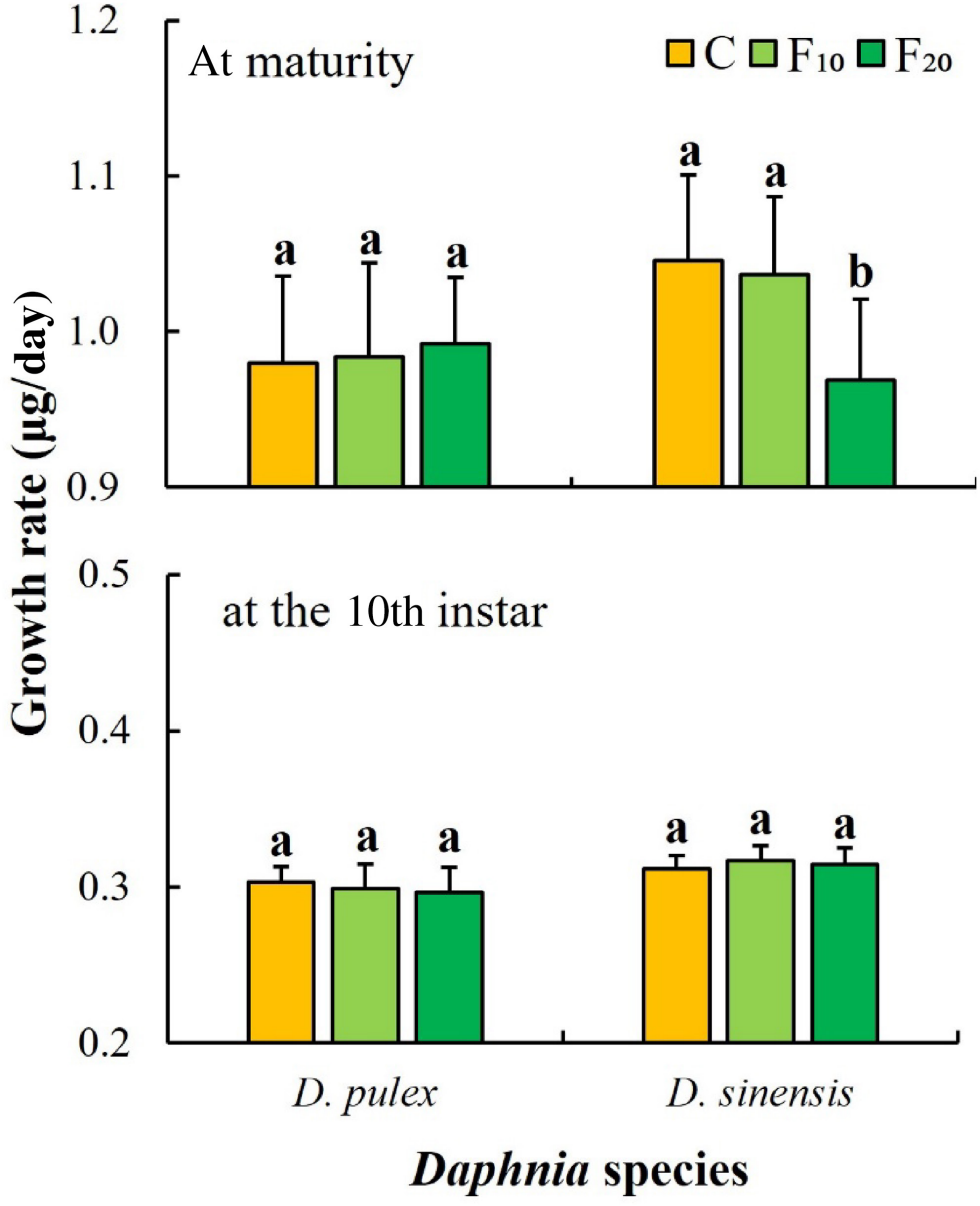
Growth rates of the two *Daphnia* species at different fish kairomone concentrations. Different letters indicate the significant differences (*p* < .05).

One‐way ANOVA showed that fish kairomones had significant effects on the growth rate at maturity of *D. sinensis*, but had no significant effects on those of *D. pulex* at maturity and at the 10th instar (Table 1). The growth rate at maturity of *D. sinensis* in the F_20_ group was significantly lower than that in the C (control) and F_10_ groups (*p* < .05, Figure 3). The growth rate at maturity and at the 10th instar of *D. pulex* did not significantly differ among the three groups (Figure 3).

**FIGURE 4:**
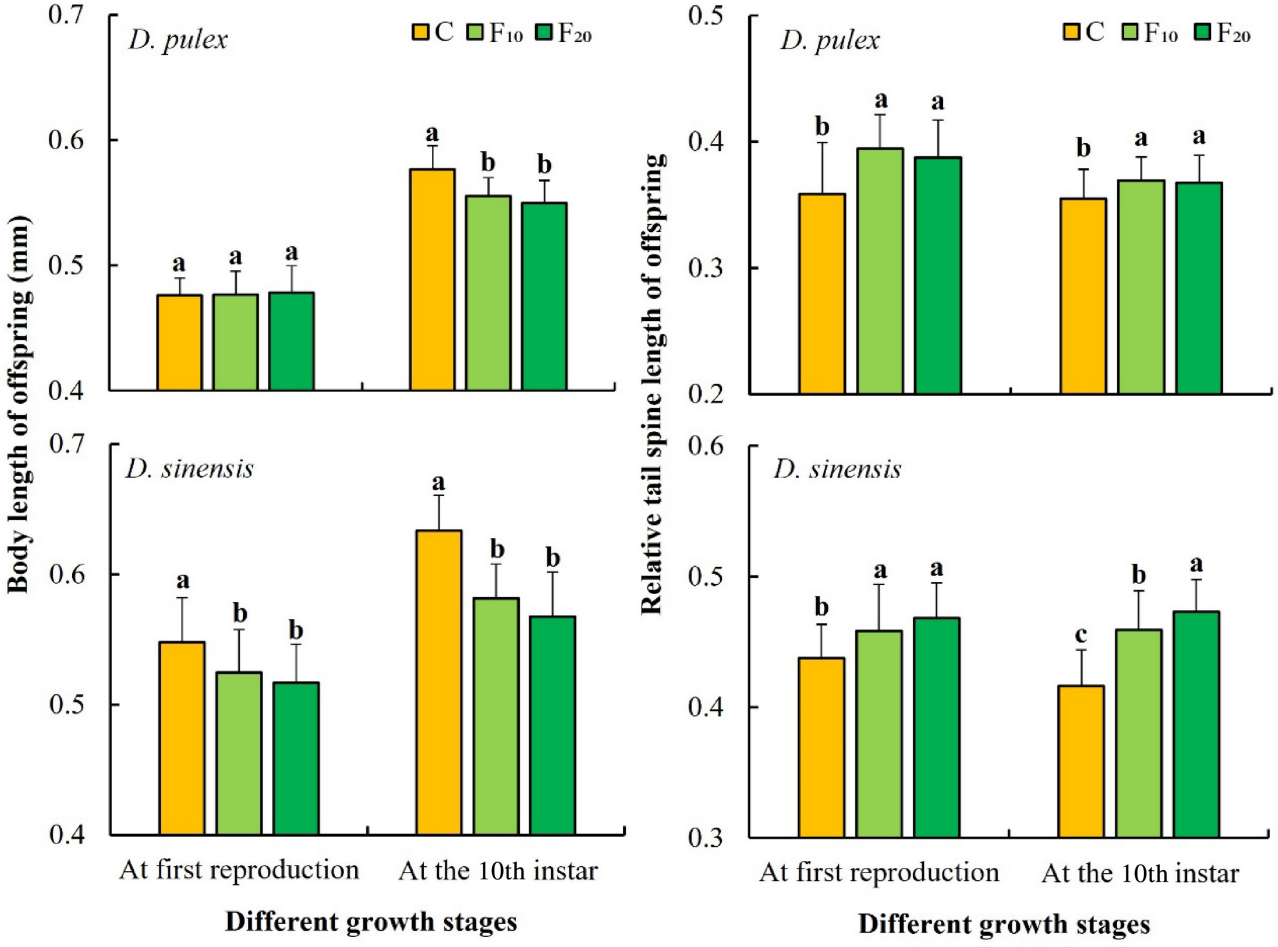
Body lengths and relative tail spine lengths of offspring at two growth stages of the two *Daphnia* species under different fish kairomone concentrations. Different letters indicate the significant differences (*p* < .05).

The effects of fish kairomones on the body length of the offspring at first reproduction of *D. sinensis* and the body length of the offspring at the 10th instar of both *D. pulex* and *D. sinensis* were significant (Table 1). The body length of the offspring at first reproduction of *D. sinensis* in the C group (control) was significantly greater than that in the F_10_ group (*p* < .05) and F_20_ group (*p* < .001). The body lengths of the offspring at the 10th instar of both *D. pulex* and *D. sinensis* in the C group (control) were significantly higher than those in the F_10_ and F_20_ groups (*p* < .001; Figure 4).

Moreover, the effects of fish kairomones on the relative tail spine lengths of the offspring at first reproduction and at the 10th instar of the two *Daphnia* species were all significant (Table 1). The relative tail spine lengths of the offspring at first reproduction and at the 10th instar of the two *Daphnia* species in the C group (control) were significantly shorter than those in the F_10_ and F_20_ groups (*p* < .05). They were also significantly shorter in the F_10_ group than in the F_20_ group (*p* < .05) for *D. sinensis* at the 10th instar (Figure 4).

### 3.2 Effects of fish kairomones on the life‐history traits of the two *Daphnia* species

**FIGURE 5:**
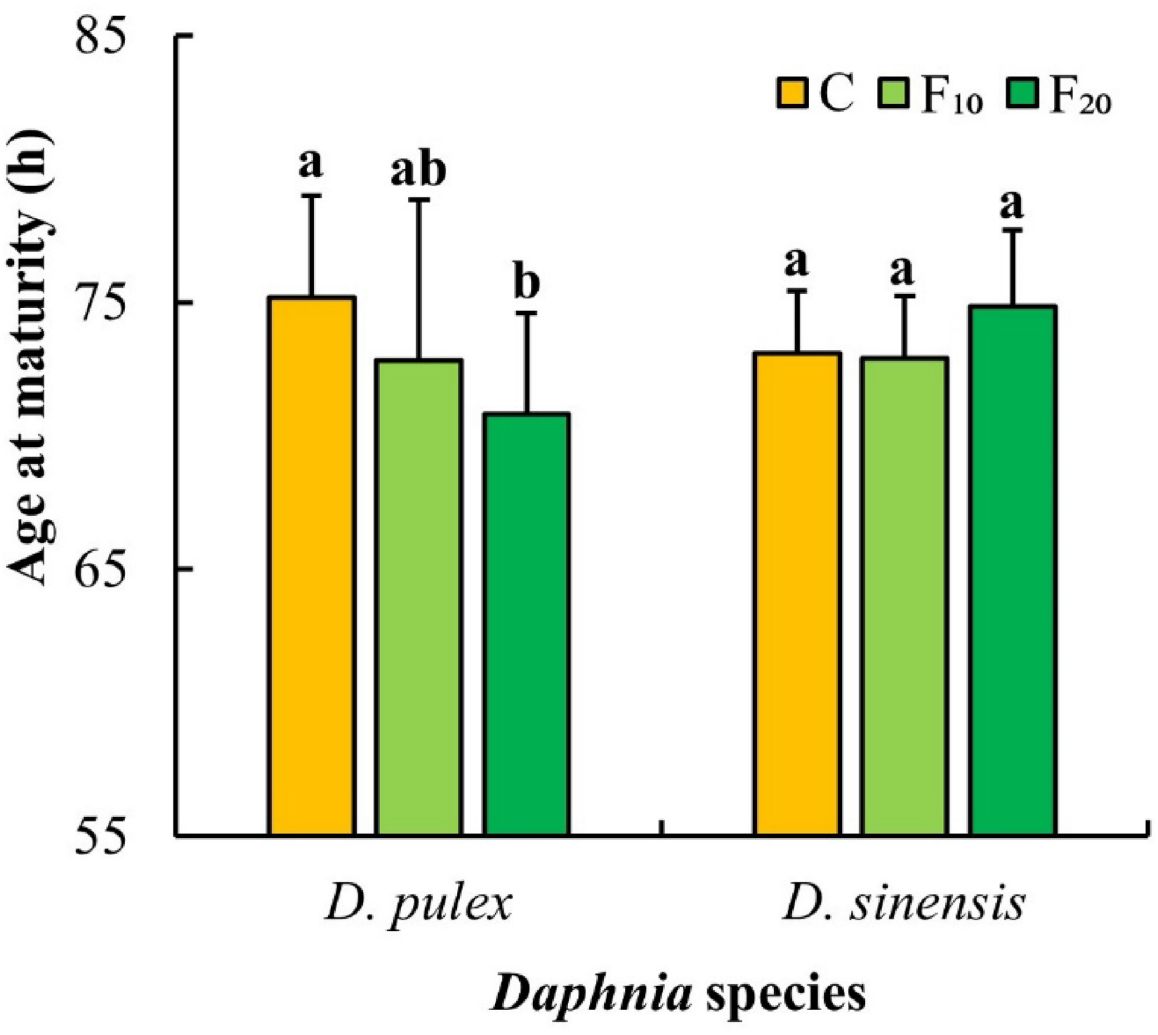
Ages at maturity of the two *Daphnia* species at different fish kairomone concentrations. Different letters indicate the significant differences (*p* < .05).

The effect of fish kairomones on the age at maturity of *D. pulex* was significant (Table 1). The age at maturity of *D. pulex* in the control group was significantly higher than that in the F_20_ group (*p* < .05, Figure 5).

**FIGURE 6:**
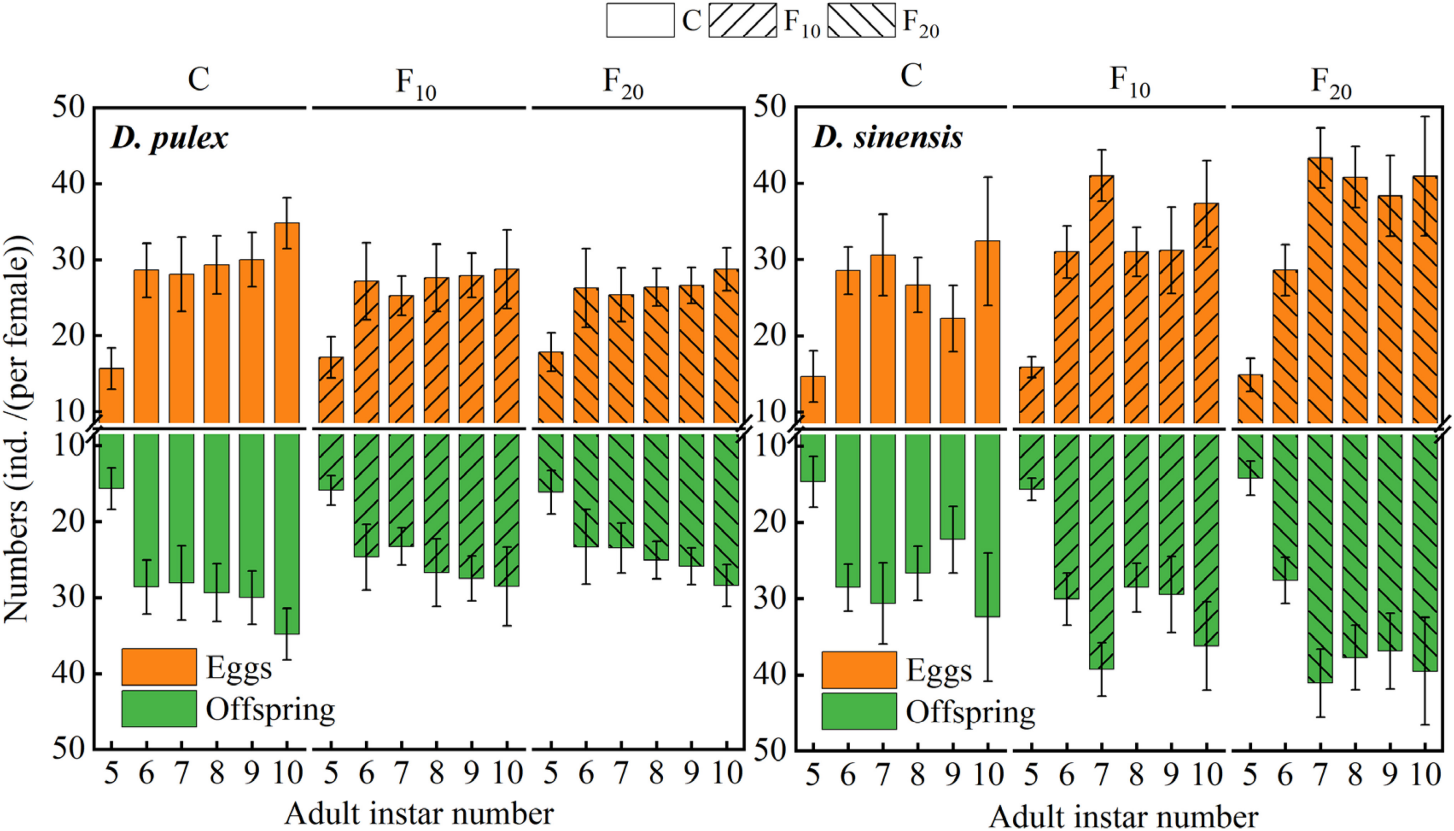
Number of eggs and offspring produced by two *Daphnia* species at different fish kairomone concentrations.

From the 6th instar, the number of eggs and offspring of *D. pulex* in the control group became higher than that in the F_10_ and F_20_ groups, whereas they became lower for *D. sinensis* from the 7th instar. The effects of fish kairomones on the total number of eggs and offspring of both *D. pulex* and *D. sinensis* were significant, whereas they did not significantly affect the number of offspring at first reproduction of the two *Daphnia* species (Table 1). Both number of eggs at first pregnancy and offspring at first reproduction in the two *Daphnia* species did not significantly differ among the three groups (Figure 6). However, both total eggs and offspring number of *D. pulex* in the C group (control) was significantly higher than that in the F_20_ (*p* < .05) besides that the total offspring number was also significantly higher in the C group (control) than in the F_10_ groups (*p* < .05), whereas both of *D. sinensis* in the C group (control) were significantly lower than that in the F_10_ and F_20_ groups (*p* < .05). Additionally, both total eggs and offspring number of *D. sinensis* were significantly lower in the F_10_ group than in the F_20_ group (*p* < .05).

**FIGURE 7:**
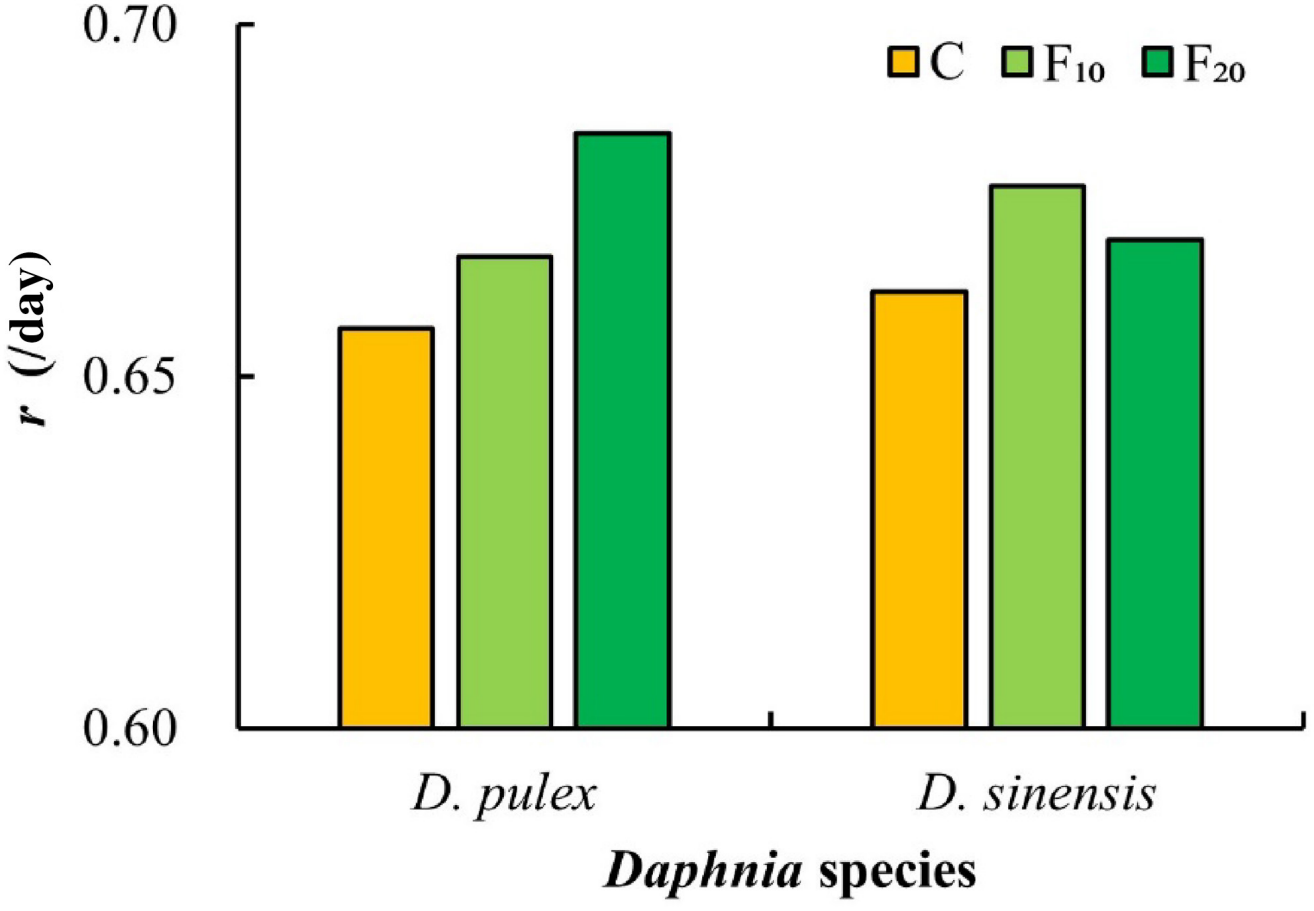
Intrinsic rate of increase (*r*) of the two *Daphnia* species at different fish kairomone concentrations.

After exposure to fish kairomones, the intrinsic rates of increase (*r*) in the two *Daphnia* species in the C group (control) were lower than those in the F_10_ and F_20_ groups (Figure 7). During the experiment, the eggs or embryos of the two *Daphnia* species disintegrated. Furthermore, males were observed in *D. pulex* offspring at a later stage of the experiment.

## 4 DISCUSSION

In this study, a typical planktivorous fish (*A. nobilis*) was selected to investigate the effects of fish kairomones on the morphological and life‐history changes of the two *Daphnia* species (*D. pulex* and *D. sinensis*), who coexisted with *A. nobilis* in Lake Chaohu. Significant differences in the inducible defenses (morphology and reproduction) of the two *Daphnia* species were observed. Many studies have also shown that inducible defenses are important means for prey to resist predation, which drives the evolution of their life‐history and behavioral and morphological traits (Castro et al., 2007; Oram & Spitze, 2013; Sperfeld et al., 2020; Tams et al., 2018; Von Elert & Loose, 1996). Usually, the changing direction and extent of *Daphnia* morphology and life‐history traits are closely related to predator species or kairomone concentrations. Moreover, previous studies have mainly utilized omnivorous fish, whereas the effects of planktivorous fish on the phenotypic plasticity of *Daphnia* species remain unclear. Therefore, our experimental results will provide reference for further exploring the adaptive evolution of *Daphnia* morphology and life‐history traits in the presence of planktivorous fish.

### 4.1 Effects of fish kairomones on the morphological changes of *Daphnia*

Morphological defenses may reduce the risk of predation because they may interfere with the catching and/or feeding processes of the predator or may prevent an attack (Diel et al., 2020). In general, visual foraging fish follow size‐selective strategies and choose larger prey when hunting (Brooks & Dodson, 1965). Reede (1995) reported that the size at maturity of *Daphnia galeata* × *hyalina* gradually decreases with increasing kairomone concentration of *Perca fluviatilis*. After exposure to kairomones released by the topmouth gudgeon (*P. parva*), bluegill sunfish (*L. macrochirus*), and *Scardinius erythrophthalmus*, the size at maturity of *D. pulex* decreases (Hanazato et al., 2001; Wilczynski et al., 2019). Similarly, in response to the kairomones of *P. fluviatilis* and *Leuciscus leuciscus*, the size at maturity of *D. galeata* decreases (Declerck & Weber, 2003; Sakwińska, 2002). The results of this study showed that the body lengths at maturity of both *D. pulex* and *D. sinensis* in groups containing *A. nobilis* kairomones (F_10_ and F_20_) were significantly shorter than that of the control group. Additionally, compared with the control group, the body length at the 10th instar of *D. pulex* was significantly shorter. The relative tail spine lengths of *D. pulex* in the two growth stages were significantly longer in the groups containing *A. nobilis* kairomones than in the control group. Thus, the inducible mechanisms of *A. nobilis* kairomones with respect to the morphology of the two *Daphnia* species differ, and the evolutionary effect of fish kairomones on the morphology (smaller body length and longer relative tail spine length) of *D. pulex* is more durable and stronger after longer exposure.

To avoid predation by fish, *Daphnia* decrease the body length at maturity or at first reproduction and produce smaller offspring (Mikulski, 2001; Pestana et al., 2013). Reede (1995) reported that the offspring size of *D. galeata* × *hyalina* linearly decreases with increasing kairomone concentration of *P. fluviatilis*. Similarly, in the presence of other fish kairomones, *D. longispina* or *D. galeata* produce smaller offspring (Castro et al., 2007; Sakwińska, 2002). The results of this study showed that the presence of *A. nobilis* kairomones led to a significant decrease in the body lengths of the offspring of the two *Daphnia* species during two growth stages (except *D. pulex* at first reproduction), whereas their relative tail spine lengths significantly increased. Engel and Tollrian (2009) observed that the tail spine length of *D. lumholtzi* affects the capture efficiency of fish and leads to predator avoidance. Therefore, it is likely that the offspring produced by *D. pulex* and *D. sinensis* exposed to *A. nobilis* kairomones can induce morphological defenses (shorter body length, longer relative tail spine length) to reduce the risk of fish predation.

### 4.2 Effects of fish kairomones on the life‐history traits of *Daphnia*

Prey can reduce the predation risk by altering life‐history traits, and these inducible defenses may counteract the predator's feeding strategy or compensate for zooplankton population loss (Diel et al., 2020). In this study, the total number of *D. sinensis* offspring significantly increased with increasing fish kairomone concentration. Some studies have shown that fish kairomones increase the number of eggs and offspring in *Daphnia* (Riessen, 1999; Sakwińska, 2002; Wilczynski et al., 2019). Kairomones released by mosquitofish (*Gambusia holbrooki*) and pumpkinseed (*Lepomis gibbosus*) induce *D. longispina* to produce more offspring (Castro et al., 2007). Moreover, Sakwińska (2002) observed that *D. galeata* produces more offspring at first reproduction in the presence of *L. leuciscus* kairomones. In this study, the number of offspring at first reproduction of the two *Daphnia* species (*D. sinensis* and *D. pulex*) did not significantly differ among the three groups. In addition, the total number of *D. pulex* offspring significantly decreased with increasing fish kairomone concentration. Other studies have also shown that the presence of fish kairomones can decrease the reproduction of *Daphnia* (Declerck & Weber, 2003; Hanazato et al., 2001; Mikulski, 2001). Similarly, *D. magna* exposed to kairomones of roach (*Rutilus rutilus*) produced fewer eggs than the control group (Burks et al., 2000). Therefore, the influence of fish kairomones on the life‐history strategies (e.g., reproduction) of *Daphnia* species depends on both fish species or kairomone concentrations and *Daphnia* species.

In the life history of cladocerans, the trade‐off between growth and reproduction varies in different stages. After an individual matures, the reduction in the investment in growth can be converted into investment in reproduction (Pijanowska et al., 2023; Rinke et al., 2008). In this study, the total number of *D. sinensis* offspring increased significantly, whereas their growth rates at maturity significantly decreased after exposure to *A. nobilis* kairomones. It suggests that a reduction in *D. sinensis* growth investment results in an increase in reproduction. Other researchers have reported that the intrinsic rate of increase (*r*) of *Daphnia* exposed to fish kairomones increases (Pestana et al., 2013; Weber, 2003). Castro et al. (2007) also reported that exposure to fish kairomones results in higher fecundity and fitness (measured as *r*) in *D. longispina*. The results of this study show that the exposure to *A. nobilis* kairomones increases the intrinsic rate of increase (*r*) in both *Daphnia* species. Furthermore, routine observations showed that some eggs in the brood chambers of the two *Daphnia* species in the fish kairomones groups had decomposed phenomenon. It is likely that *Daphnia* species allocate part of their energy to regulate morphological changes under the induction of fish kairomones such that there is not enough energy for the development of eggs or embryos. In contrast to *D. sinensis*, both the total number of offspring of *D. pulex* significantly decreased, and the age at maturity of *D. pulex* significantly shortened. Therefore, we hypothesized that both *D. pulex* and *D. sinensis* may allocate energy resources in different ways to regulate morphological (e.g., body length, spine length) or life‐history (e.g., reproduction) changes to avoid predation when exposed to *A. nobilis* kairomones.

Ke and Huang (2009) thought that the phenotypic plasticity of *Daphnia* was a result of adaptive evolution under long‐term interactions between *Daphnia* populations and predators. In Lake Chaohu, the two *Daphnia* species dominated in different seasons (*D. pulex* in March and April whereas *D. sinensis* in May and June) (Deng et al., 2008), and there may be difference in the composition and structure of predators. In this study, the reproductive output (offspring numbers) of the two *Daphnia* species exposed to *A. nolilis* kairomones had a significant difference, and it might be related to long‐term adaptive evolution under different predators (including *A. nolilis*) except interspecific difference. Seda and Petrusek (2011) observed also that the pressures of *Chaoborus* and fish resulted in rapid microevolutionary changes of antipredator mechanisms in *D. longispina* together.

## 5 CONCLUSIONS

Our study shows that *Daphnia* can develop inducible defenses (morphological defenses and life‐history defenses) by regulating the growth and reproductive resources and adapting to a prolonged predation risk through changes in the phenotypic plasticity. In the presence of *A. nobilis* kairomones, a significantly smaller body length at maturity, smaller body length of offspring at the 10th instar stage, and larger relative tail spines of offspring of the two *Daphnia* species were observed. However, differences in other morphological and life‐history traits were notable between the two *Daphnia* species. Compared with *D. sinensis*, a significantly higher relative tail spine length and earlier age at maturity of *D. pulex* after exposure to *A. nobilis* kairomones were observed. Additionally, the total number of *D. sinensis* offspring in the groups containing *A. nobilis* kairomones was significantly higher than that in the control group, whereas it was significantly lower for *D. pulex*, suggesting a contradictory strategy for the reproduction of the two *Daphnia* species when exposed to *A. nobilis* kairomones. These results suggest that the two *Daphnia* species have different inducible defense strategies (e.g., morphological and life‐history traits) during prolonged exposure to *A. nobilis* kairomones, and their offspring also develop morphological defenses to reduce the risk of fish predation. It will provide reference for further exploring the adaptive evolution of *Daphnia* morphology and life‐history traits in the presence of planktivorous fish.

## AUTHOR CONTRIBUTIONS

**Qide Jin:** Data curation (equal); formal analysis (equal); investigation (lead); methodology (equal); software (equal); visualization (lead); writing – original draft (lead); writing – review and editing (equal). **Yeping Wang:** Investigation (supporting). **Kun Zhang:** Formal analysis (equal); methodology (equal); software (lead); validation (supporting). **Guoqing Li:** Investigation (supporting). **Yanan Chen:** Investigation (supporting). **Yujuan Hong:** Investigation (supporting). **Hanxue Cheng:** Investigation (supporting). **Daogui Deng:** Conceptualization (lead); data curation (equal); funding acquisition (lead); methodology (equal); project administration (equal); supervision (equal); writing – original draft (supporting); writing – review and editing (equal).

## CONFLICT OF INTEREST STATEMENT

The authors declare no conflicts of interest.

## Data Availability

All data used for this study have been deposited in Dryad Digital Repository (https://datadryad.org/stash/share/9myLce0rZFPsLZmVP3zLZyHJA5j1kopeCmbTPGhOQdA).
